# ROS-Neuro: An Open-Source Platform for Neurorobotics

**DOI:** 10.3389/fnbot.2022.886050

**Published:** 2022-05-10

**Authors:** Luca Tonin, Gloria Beraldo, Stefano Tortora, Emanuele Menegatti

**Affiliations:** ^1^Intelligent Autonomous Systems Laboratory, Department of Information Engineering, University of Padova, Padua, Italy; ^2^Padova Neuroscience Center, University of Padova, Padua, Italy; ^3^Institute of Cognitive Sciences and Technologies, National Research Council, Rome, Italy

**Keywords:** ROS, ROS-Neuro, neural interface, brain-machine interface, neurorobotics

## Abstract

The growing interest in neurorobotics has led to a proliferation of heterogeneous neurophysiological-based applications controlling a variety of robotic devices. Although recent years have seen great advances in this technology, the integration between human neural interfaces and robotics is still limited, making evident the necessity of creating a standardized research framework bridging the gap between neuroscience and robotics. This perspective paper presents Robot Operating System (ROS)-Neuro, an open-source framework for neurorobotic applications based on ROS. ROS-Neuro aims to facilitate the software distribution, the repeatability of the experimental results, and support the birth of a new community focused on neuro-driven robotics. In addition, the exploitation of Robot Operating System (ROS) infrastructure guarantees stability, reliability, and robustness, which represent fundamental aspects to enhance the translational impact of this technology. We suggest that ROS-Neuro might be the future development platform for the flourishing of a new generation of neurorobots to promote the rehabilitation, the inclusion, and the independence of people with disabilities in their everyday life.

## 1. Introduction

The last few years have seen a growing interest in the topic of neural human-machine interfaces as a novel—potentially groundbreaking—interaction modality between users and robotic devices. In these interfaces, neurophysiological signals are acquired in real-time [e.g., from electroencephalography (EEG) or from electromyography (EMG)], processed with minimum delay, and translated into commands for the external actuators. Based on this workflow, researchers have demonstrated the feasibility and the potentiality of this innovation, in particular for those people suffering from severe motor disabilities (Kennedy and Bakay, 1998; Hochberg et al., 2012; Aflalo et al., 2015; Chaudhary et al., 2016; Tonin and Millán, 2021). For instance, the latest advances in the brain-machine interface (BMI) showed the possibility to exploit brain signals (acquired with invasive or non-invasive techniques) to control telepresence robots, powered wheelchairs, robotic arms, and upper/lower-limb exoskeletons (Iez et al., 2010; Leeb et al., 2013, 2015; Liu et al., 2017, 2018; Edelman et al., 2019). In parallel, systems relying on residual motor functions demonstrated that EMG signals can be re-interpreted and used to precisely drive robotic arms in amputees (Farrell and Weir, 2008; Castellini et al., 2009; Cipriani et al., 2011; Borton et al., 2013; Parajuli et al., 2019), to initiate the walking pattern in lower-limb exoskeletons (Sylos-Labini et al., 2014; De Luca et al., 2019) or to support reaching and grasping tasks with upper-limb exoskeletons (Batzianoulis et al., 2017, 2018; Betti et al., 2018).

However, despite such an emerging and promising trend, the full potential of the field is still unrevealed. Among the multifaceted and multidisciplinary aspects belonging to the neurorobotics challenge, herein we propose an engineering perspective on the development of neural driven robotic devices. In this regard, we highlight three current drawbacks that are conceptually and technically narrowing the field: first, the community suffers from the lack of a common development platform to spread the latest advances, to consolidate prototypes, and to compare results among different research groups. Second, there has been an abundance of home-made solutions that inevitably led to a heterogeneity of technical approaches to the same problems and to an absence of standards, making the reuse of already developed and well-tested code problematic. Finally, recent research trends keep considering robotic devices as mere passive actuators of users' intentions by mostly neglecting the potential benefits of including robotic artificial intelligence in the decoding workflow. Furthermore, we speculate that the lack of technical tools (e.g., a common development ecosystem) might also conceptually affect the direction of the current neurorobotics research by slowing down the necessary integration between neural interfaces and robotics. It is worth mentioning that a variety of open-source platforms already exists in the neurorobotics field to acquire, process, and decode neurophysiological signals (e.g., LSL, BCI2000, OpenViBE, TOBI Common Implementation Platform, BioSig, BCILAB, BCI++, xBCI, BF++, PMW, and VETA Brunner et al., 2012; Stegman et al., 2020). Although each software has specific features and advantages, they only partially face all the aforementioned challenges. Furthermore, to the best of our knowledge, neither of them explicitly targets the integration of robotic platforms nor do they provide out-of-the-box solutions to directly interact with external devices.

In the current scenario, we firmly believe in the urgency of a common and open-source research framework for the future development of the neurorobotic field. Hence, we spotlight Robot Operating System (ROS)-Neuro, the first middleware explicitly devised to treat the multidisciplinary facets of neurorobotics with the same level of importance, to promote a holistic approach to the field, and to foster the research of a new generation of neural driven robotic devices.

## 2. ROS-Neuro middleware

### 2.1. Overview

ROS-Neuro has been designed to represent the first open-source neurorobotic middleware that places human neural interfaces and robotic systems at the same conceptual and implementation level. ROS-Neuro is an extension of ROS that for many years is considered the standard platform for robotics (Quigley et al., 2009). One of the strengths of ROS is its modularity and the possibility for different research groups to develop stand-alone components all relying on the same standard communication infrastructure. A similar requirement is a cornerstone for the workflow of any closed-loop neural interface where—for instance—acquisition, processing, and decoding methods should run in parallel in order to provide a continuous/discrete control signal to drive the robotic device. ROS-Neuro not only exploits such modular design but also provides several standard interfaces to acquire neurophysiological signals from different commercial devices to process EEG and EMG signals with traditional methods and to classify data with common machine learning algorithms. As in the case of ROS, the aim of ROS-Neuro is to allow the development of neurorobotic applications among different research groups as well as the possibility to easily compare heterogeneous methodological approaches and to rely and evaluate solutions proposed by others. This is guaranteed by its multi-process architecture where several stand-alone executables can coexist and can communicate through the provided network infrastructure. Moreover, each of these processes can be easily exchanged between research groups with the only requirement of sharing the same interface. The concept of ROS-Neuro has been introduced for the first time in Beraldo et al. (2018b) and in the following years, authors implemented and carefully tested packages to acquire, record, process, and visualize EEG and EMG data (Tonin et al., 2019; Beraldo et al., 2020). The aim of this contribution is to present ROS-Neuro to the community by providing a description of its main features and potentialities.

### 2.2. Abstraction, Modularity, and Parallel Architecture

Robotic applications and human neural interfaces share several similarities in the technical and implementation workflow. As robotics is traditionally based on the interactions between perception and planning and action, neural interfaces rely on the acquisition, processing, and classification closed-loop where the human plays the twofold role of generating the input signals and monitoring (as well as adapting to) the results of the decoding. Tonin and Millán (2021). ROS-Neuro generalizes such an architecture by providing modules to gather neurophysiological signals (rosneuro_acquisition package), to record the acquired data (rosneuro_recorder), to process and decode it (rosneuro_buffers, rosneuro_filters, rosneuro_processing), and to finally infer the intention of the user (rosneuro_decisionmaking). As in the case of the packages available in the ROS ecosystem, these modules represent generic interfaces that neither depends on specific hardware devices nor on particular processing methods. For instance, rosneuro_acquisition package is designed to work with plugins that can support different EEG/EMG devices and that can be independently developed (and shared) by any research group according to their needs. However, it is worth mentioning that ROS-Neuro already provides plugins that interface with the most used commercial acquisition systems (e.g., g.Tec, BioSemi, ANTNeuro, Cognionics). Similarly, packages like rosneuro_buffers and rosneuro_filters implement widely commonly used methods to process neural data such as spatial filters, DC removal algorithms, and windowing that can be easily extended and integrated with custom solutions provided by researchers. Table 1 lists the acquisition systems (hardware devices and software platforms) compatible with ROS-Neuro and the supported file formats to store the acquired data. Furthermore, the filters, buffers, and the application scope provided by ROS-Neuro are reported.

**Table 1:** List of acquisition devices and platforms currently compatible with the rosneuro_acquisition package and the file formats supported by the rosneuro_recorder.

**Hardware**	**Company**	**Driver**	**Plugin**	**Status**
**rosneuro_acquisition**				
BioSemi ActiveTwo	BioSemi	free	rosneuro::EGDDevice	Tested
MindWave Headsets	Neurosky	free	rosneuro::EGDDevice	Untested
Bittium NeurOne	Bittium	free	rosneuro::EGDDevice	Untested
g.USBamp	g.Tec	proprietary	rosneuro::EGDDevice	Tested
g.NEEDaccess	g.Tex	proprietary	rosneuro::EGDDevice	Untested
BitBrain EEG	BitBrain	proprietary	rosneuro::EGDDevice	Untested
DSI-24	Wearable Sensing	proprietary	rosneuro::EGDDevice	Tested
CGX Quick-20	Cognionics	proprietary	rosneuro::EGDDevice	Tested
eego sport and mylab	AntNeuro	proprietary	rosneuro::EGDDevice	Tested
Ultracortex Mark IV	OpenBCI	free	rosneuro::LSLDevice	Tested
LabStreaming layer	/	free	rosneuro::LSLDevice	Tested
Tobi Interface A	/	free	rosneuro::EGDDevice	Untested
General data format (GDF) file	/	free	rosneuro::EGDDevice	Tested
BioSemi data format (BDF) file	/	free	rosneuro::EGDDevice	Tested
**File format**	**Company**	**Driver**		**Status**
**rosneuro_recorder**				
General data format (GDF)	/	free		Tested
BioSemi data format (BDF)	BioSemi	free		Tested
**Filter**	**Type**		**Class**	**Status**
**rosneuro_filters**				
DC removal	Temporal		rosneuro::Dc < T>	Tested
Common Average Reference	Spatial		rosneuro::Car < T>	Tested
Laplacian derivation	Spatial		rosneuro::Laplacian < T>	Tested
Blackman	Windowing		rosneuro::Blackman < T>	Tested
Flattop	Windowing		rosneuro::Flattop < T>	Tested
Hamming	Windowing		rosneuro::Hamming < T>	Tested
Hann	Windowing		rosneuro::Hann < T>	Tested
**Buffer**	**Type**		**Class**	**Status**
**rosneuro_buffers**				
RingBuffer	FIFO		rosneuro::RingBuffer < T>	Tested
**Application**	**Type**			**Status**
**rosneuro_visualizer**				
neuroviz	Temporal scope			Tested

*The table also provides the filters and buffers available in the rosneuro_filters and rosneuro_buffers packages. It is worth noticing that both filters and buffers can be easily concatenated via configuration file [please refer to FilterChain in Robot Operating System (ROS)]. Finally, neuroviz application is listed—the electroencephalography (EEG)/electromyography (EMG) scope provided by the rosneuro_visualizer package. In the last column, Untested status means that the related hardware is technically supported by the plugin but it was not possible to test it*.

**Figure 1:**
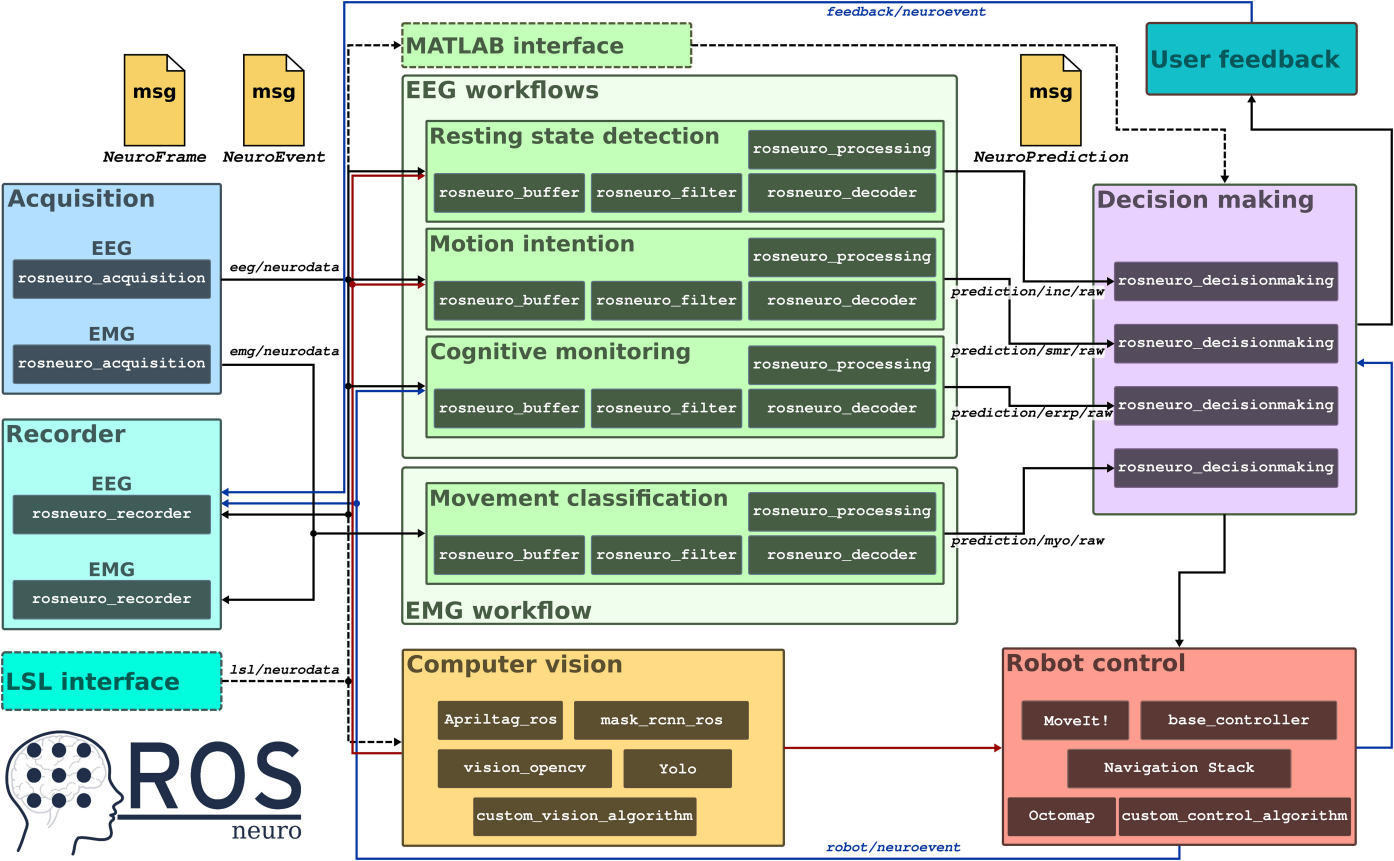
A schematic representation of a hybrid, multi-process implementation of a neural interface with Robot Operating System (ROS)-Neuro. Two acquisition systems are used in parallel to acquire (and record) EEG and EMG data (blue and cyan boxes). An additional interface can be added to record the data stream from the LSL device (dashed cyan box). Data is made available in the eeg/neurodata and emg/neurodata communication channels as NeuroFrame messages to all the other modules. In the example, four different workflows work in parallel (green boxes) to detect resting state, motion intention, to monitor the behavior of the system from EEG signals, and to classify residual muscular activity from EMG data. An additional processing module can be added by exploiting the ROS-Neuro MATLAB interface (dashed green box). The output of the processing workflows is published as NeuroPrediction messages in the prediction/*/raw. A decision making module (purple box) reads the predicted outputs and generates a proper control signal for the robotic device. Such a signal can be also used to provide feedback to the user. In parallel, computer vision algorithms and ROS navigation packages (red and yellow boxes) not only take care of controlling the robot but also provide environmental information for the EEG workflows (red and blue arrows).

Another feature of ROS-Neuro is the possibility to conveniently implement parallel pipelines with the minimum developing effort. This is of particular interest for many emerging aspects of hybrid neural interfaces. On the one hand, these interfaces are designed to simultaneously acquire, process, and fuse together heterogeneous neurophysiological signals from several sources [e.g., EEG, EMG, electrooculography (EOG)] in order to improve the robustness of the whole system (Müller-Putz et al., 2011). On the other, they can rely on different processing workflows to decode concurrent tasks performed by the user. In both cases, ROS-Neuro already exploits the ROS optimized communication infrastructure and it can rely on built-in solutions to synchronize and align data streams from different processes (e.g., hardware-based trigger) (Bilucaglia et al., 2020). This enormously facilitates the implementation of interfaces where—for instance—multiple acquisition processes are instantiated to simultaneously gather EEG and EMG signals (Figure 1). Then, specific processing may be applied to EEG in order to decode the intention of the user to reach an object with a robotic arm or an upper-limb exoskeleton; at the same time, residual muscular activity may be exploited to distinguish the type of grasping. Furthermore, brain signals can be also analyzed in conjunction with environmental information in order to recognize potential erroneous actions performed by the neuroprosthesis.

### 2.3. Standard Messages and Communication

The rapid growth of the neurorobotics field, and in particular, of human neural interfaces has led to the heterogeneity of technical solutions. In this scenario, one of the main limitations of current developing frameworks is the custom approaches to sharing information between the different modules composing the closed-loop implementation of neural interfaces. Traditionally, each research group relies on its own data structures to represent neurophysiological data and custom-made network infrastructures to implement the communication between the several processing steps. Such a lack of a common approach strongly downplays the impact of the technology by limiting the possibility to share developing tools, to exploit solutions already implemented, and to replicate results achieved by different research groups.

ROS-Neuro provides standard messages to exchange data structures between the modules usually implemented within neurorobotics applications. Moreover, messages are available to all modules by the ROS network infrastructure-based peer-to-peer communication mechanisms. Data acquired by the rosneuro_acquisition is streamed as NeuroFrame messages within the ecosystem (Figure 1), where several modules can subscribe to the stream at the same time and concurrently process the messages in order to extract and decode heterogeneous features from neurophysiological signals. Similarly, the output of the decoder is translated into NeuroPrediction messages that can be exploited to directly control the robotic application or to be further processed. Furthermore, it is worth mentioning that ROS allows to quickly extend the interface of any message without the need for coding in order to handle specific, application-related requirements. As a consequence, ROS-Neuro not only offers the possibility to conveniently compare different methodological approaches even during closed-loop operations but also to effortlessly distribute implementation solutions among different research groups with the only requirement of providing the standard message interface.

### 2.4. Robotic Devices

The straightforward integration between neural interfaces and external actuators is the most evident advantage of ROS-Neuro middleware. Traditionally, the inclusion of robotic devices has been considered a pure technical challenge, and thus, a variety of home-made solutions has been adopted to deliver the output of the neural interface to the robot ecosystem. However, the drawback of this approach is twofold: first, from an engineering perspective, custom solutions are often not optimized and efficient with the consequence of an increased risk of technical faults. Second, the communication stream between neural interfaces and robotic devices is usually limited to a single uni-dimensional control signal. This definitely narrows the research on new human-machine interaction (HMI) modalities and the introduction of bidirectional communication with the robot to enhance the robustness and the reliability of the whole system. For instance, a robot's intelligence may provide information about the operational context to the neural interface in order to modulate the velocity of the decoder response, thus facilitating the control or preventing the delivery of an erroneous command according to the current situation. Thus, the level of autonomy of the neurorobotic device may be changed in the case, for example, a smart wheelchair crosses a narrow passage or a robotic hand attempts to grasp an unusual-shaped object (Figure 1).

By construction, ROS-Neuro explicitly provides such a common and bidirectional communication between the neural interface workflow and the robotic intelligence by exploiting the ROS ecosystem and the several packages already available in the ROS community. Furthermore, the reliability and robustness of the communication is guaranteed by the ROS network infrastructure by reducing the likelihood of technical shortcomings and malfunctions.

## 3. Evaluation of ROS-Neuro: the Cybathlon Event

ROS-Neuro has been evaluated by using different hardware devices (e.g., a variety of commercial EEG/EMG amplifiers and various robotic platforms Beraldo et al., 2018a,b, 2020; Tonin et al., 2019) during several experiments. In all cases, ROS-Neuro demonstrated its flexibility, reliability, and robustness. However, the most critical stress test for ROS-Neuro has been the usage for the Cybathlon events (Wolf and Riener, 2018). Cybathlon is the first neurorobotic championship where several international teams from all over the world competed in different disciplines: from races with lower and upper limb prostheses to races with wheelchairs and exoskeletons. The ultimate goal of Cybathlon is to foster the research and development of daily-life solutions for people with disabilities. In this context, one of the most challenging disciplines was the BCI Race^1^ where pilots with a severe motor disability (i.e., inclusion criteria ASIA-C) exploited a non-invasive BMI to control an avatar on the screen during a virtual race. Authors participated in the Cybathlon BCI Series 2019 and the Cybathlon 2020 Global Edition with the WHi Team composed of researchers from the University of Padua (Italy). In these periods and in the related longitudinal training of the pilot, ROS-Neuro has been extensively used and tested. In both editions, the WHi Team won the gold medal by awarding the race records. Most importantly, ROS-Neuro was confirmed to be reliable and robust during the whole training and, especially, in the demanding conditions of the event. Neither technical faults nor difficulties or glitches during the interface with the official Cybathlon infrastructure (for connecting to the virtual race) have been reported. We speculate that the efficiency, the flexibility, and the performance of ROS-Neuro were one of the key reasons (among others) for the success of the WHi Team at the Cybathlon.

## 4. Discussion

Recent evidence in literature highlighted the importance of reconsidering the current approach to neurorobotics in order to enhance the reliability of neural driven robotic devices, and thus, foster the translational impact and the daily usage of the technology (Perdikis et al., 2018; Perdikis and Millán, 2020; Tonin and Millán, 2021). In particular, the research community started following a more holistic approach by investigating the mutual interactions between the actors of the system, i.e., the user, the decoder, and the robotic device. For instance, several studies have demonstrated the key role of mutual learning between user and decoder to facilitate the acquisition of BMI skills and enhance the reliability of BMI-driven devices (Perdikis and Millán, 2020). Similarly, it has been shown that a neural interface explicitly designed to promote the interaction between user and robotic intelligence can support a more natural and efficient control of the device (Tonin et al., 2020). In this scenario, we speculate that ROS-Neuro might offer the technical counterpart of this new research direction by not only allowing to develop the neural interface workflow and the robotic intelligence within the same ecosystem but also by guaranteeing high performance and strong robustness of the whole application.

Although we previously pinpointed ROS-Neuro features with respect to the current platforms available in the community, it is worth mentioning that it should not be considered a direct competitor. Indeed, ROS-Neuro represents uniqueness in the neurorobotics field with the explicit aim of integrating neural interfaces and robotics by exploiting the advantages of both fields. Furthermore, current development frameworks to acquire neural signals can easily be included in the ROS-Neuro infrastructure, for instance, plugin to connect LSL is already implemented and available in the public repository to incorporate external information streams into the ROS-Neuro ecosystem.

ROS-Neuro is distributed as an open-source project, and it is available on GitHub^2^. As in the case of ROS, the success of ROS-Neuro strictly depends on the creation of a wide community disseminating the latest developments and including the multidisciplinary needs of the different research groups. It is our opinion that ROS-Neuro represents the only way to achieve a robust and flexible ecosystem, to review and evaluate alternative approaches, and, finally, to boost neurorobotics technology. ROS-Neuro supports the development of packages in C++ and Python, and we acknowledge that this might hinder the approach to the platform, especially if researchers are used to working with GUI-based software (e.g., OpenVibe). For this reason, ROS-Neuro already provides a MATLAB interface (rosneuro_matlab) in order to facilitate the integration with toolboxes widely spread in the community and to mitigate the effort of those people not used to such programming languages. Nevertheless, we consider that this is a small price to pay in comparison with the advantages in terms of reliability, performance, and integration that ROS-Neuro can offer.

Finally, the current version of ROS-Neuro is fully based on ROS 1 LTS (ROS Noetic Ninjemys)^3^, and thus, it works on Ubuntu Linux operating systems only. However, in a few years, the community started the development of ROS 2 that—among several changes—is the first multi-platform version of ROS (i.e., on Ubuntu Linux, MacOS, and Windows 10). The transition of ROS-Neuro from ROS 1 to ROS 2 has already been scheduled to expand the base of potential users of ROS-Neuro. Nevertheless, the effort to develop and maintain both versions can be demanding, and it would be beneficial to have the support of the whole community.

In conclusion, we firmly believe that ROS-Neuro might be the future development platform for neurorobotics. Furthermore, as in the case of ROS, it might represent the starting point for the creation of a flourishing research community to foster the translational impact of neurorobotics technology.

## Data Availability Statement

Publicly available code was reported in this study. This code can be found here: GitHub, https://github.com/rosneuro.

## Author Contributions

LT and EM conceived the idea of ROS-Neuro. All authors wrote, reviewed, and approved the final manuscript.

## Funding

Part of this work was supported by MUR (Italian Minister for University and Research), under the initiative Departments of Excellence (Law 232/2016), and by the Department of Information Engineering, University of Padova, under the BrainGear project (TONI_BIRD2020_01).

## Conflict of Interest

The authors declare that the research was conducted in the absence of any commercial or financial relationships that could be construed as a potential conflict of interest.

## Publisher's Note

All claims expressed in this article are solely those of the authors and do not necessarily represent those of their affiliated organizations, or those of the publisher, the editors and the reviewers. Any product that may be evaluated in this article, or claim that may be made by its manufacturer, is not guaranteed or endorsed by the publisher.

## Footnotes

^1^ https://cybathlon.ethz.ch/en/event/disciplines/bci

^2^ https://github.com/rosneuro

^3^ https://www.ros.org/
